# Etiology, Clinical Course, and Outcomes of Pneumonia in the Elderly: A Retrospective and Prospective Cohort Study in Thailand

**DOI:** 10.4269/ajtmh.20-1393

**Published:** 2021-05-03

**Authors:** Mayada Osman, Weerawat Manosuthi, Jaranit Kaewkungwal, Udomsak Silachamroon, Chayasin Mansanguan, Supitcha Kamolratanakul, Punnee Pitisuttithum

**Affiliations:** 1 Faculty of Tropical Medicine, Mahidol University, Ratchathewi, Bangkok, Thailand;; 2 Department of Medicine, Bamrasnaradura Infectious Diseases Institute, Nonthaburi, Thailand

## Abstract

Pneumonia is a leading cause of hospitalization and death among elderly adults. We performed a retrospective and prospective observational study to describe the etiology, clinical course, and outcomes of pneumonia for patients 60 years and older in Thailand. We enrolled 490 patients; 440 patients were included in the retrospective study and 50 patients were included in the prospective study. The CURB-65 score and a modified SMART-COP score (SMART-CO score) were used to assess disease severity. The median patient age was 80 years (interquartile range, 70–87 years); 51.2% were men. *Klebsiella pneumoniae* (20.4%) and *Pseudomonas aeruginosa* (15.5%) were the most common causative agents of pneumonia. A significant minority (23%) of patients were admitted to the intensive care unit (ICU), and mortality among this subset of patients was 45%. Most patients (80.8%) survived and were discharged from the hospital. The median duration of hospitalization was 8 days (interquartile range, 4–16 days). In contrast, 17.6% of patients died while undergoing care and 30-day mortality was 14%. Factors significantly associated with mortality were advanced age (*P* = 0.004), male sex (*P* = 0.005), multiple bacterial infections (*P* = 0.007; relative risk [RR], 1.88; 95% confidence interval [CI], 1.19–2.79), infection with multi-drug-resistant/extended-spectrum B-lactamase-producing organisms (*P* < 0.001; RR, 2.82; 95% CI, 1.83–4.85), ICU admission (*P* < 0.001; RR, 1.8; 95% CI, 1.4–2.3), and complications of pneumonia (*P* < 0.001; RR, 2.5; 95% CI, 1.8–3.4). Patients with higher SMART-CO and CURB-65 scores had higher rates of ICU admission and higher 30-day mortality rates (*P* < 0.001). These results emphasize the importance of Gram-negative bacteria, particularly *K. pneumoniae* and *P. aeruginosa*, as major causes of pneumonia among the elderly in contrast to other reports, *Streptococcus pneumoniae* is a common cause of pneumonia among elderly individuals worldwide. The SMART-COP and CURB-65 scores were developed to assess pneumonia severity and predict mortality of young adults with pneumonia. Few studies have examined the appropriateness of these scores for elderly patients with multiple comorbidities. A limited number of studies have used modified versions of these scores among elderly individuals. We found that Gram-negative bacteria has a major role in the etiology of pneumonia among elderly individuals in Southeast Asia. A significant proportion of elderly individuals with low CURB-65 scores were admitted to the hospital, indicating that hospital admission may reflect fragility among elderly individuals with low CURB-65 scores. The modified SMART-COP score (SMART-CO score) sufficiently predicted intensive care unit admission and the need for intensive vasopressor or respiratory support. A SMART-CO score ≥ 7 accurately predicted 30-day mortality.

## INTRODUCTION

Pneumonia is one of the most common infectious diseases in clinical practice and a frequent cause of hospital admission and mortality worldwide. For elderly individuals, factors such as weakened or suppressed immunity, comorbidities, diminished cough reflex, and poor functional status contribute to the increased incidence of pneumonia compared with that of younger adults.^1,2^

Among the elderly, the presentation, etiology, clinical course, and outcomes of pneumonia may differ compared with those of younger adults. The etiological agents causing pneumonia in elderly patients vary geographically.^3–5^ *Streptococcus pneumoniae* is the most common cause of community-acquired pneumonia (CAP) in Western European countries^6^ and developing countries.^7,8^ In contrast, in others Asian countries, Gram-negative bacteria are common causes of pneumonia among the elderly and result in high mortality rates.^2,9^ For many elderly patients with pneumonia, the etiological agent is difficult to identify because of their inability to expectorate or use of empiric antimicrobial therapy.

Rapid progression of pneumonia in the elderly can predispose these patients to severe disease. Elderly individuals are at high risk for infection by multidrug-resistant organisms, and the inability to treat these patients with common antibiotics can result in more severe outcomes. Fragile patients with underlying comorbidities are more likely to require intensive care unit (ICU) admission and intensive respiratory or vasopressor support. Mortality rates are increased for elderly patients with pneumonia admitted to ICUs,^3^ reaching up to 55.9% in some Asian countries.^9^ Older age (older than 60 years) was previously shown to be an independent risk factor associated with pneumonia severity.^10^ Chronic renal disease and electrolyte disturbances were also identified as risk factors for severe pneumonia in the elderly.^11^ Older adults with pneumonia require a long recovery period; one study showed that 60 days were required for elderly individuals with underlying respiratory diseases to recover from an episode of pneumonia.^12^ Previous hospital admission for pneumonia was associated with a higher risk of recurrent admission.^13^ One systemic review and meta-analysis found that 30-day readmission rates ranged from 7.8% to 19.3%.^14^ Furthermore, recurrent pneumonia is common among the elderly.^15^

Many scoring systems have been developed to predict pneumonia severity,^16^ mortality,^17^ ICU admission, and the need for intensive respiratory or vasopressor support.^18^ These scoring systems can help clinicians in developing countries with limited resources by enabling early detection of seriously ill patients who require special care or ICU admission. Delays in ICU admission are associated with poor outcomes.^19^ However, clinical judgment is the gold standard used to determine disease severity. During this study, we used the CURB-65 score (developed by the American Thoracic Society/Infectious Diseases Society of America), which is widely recommended for the assessment of patients with CAP.^16^ We also used a modified SMART-COP score (originally developed by Australian researchers),^18^ which has performed well in emergency settings in developing countries.^20^ Other modified versions have been used by primary care physicians^21^ and showed high sensitivity in tropical areas.^22^ These two scoring systems were used to assess disease severity. We also analyzed the relationships of these scores with patient ICU admission, the need for intensive respiratory support, and 30-day mortality.

Because of the significant impact of pneumonia on older adults and the lack of data regarding the causative agents of CAP among an elderly Asian population, we aimed to describe the etiology, clinical course, and outcomes of pneumonia for adults 60 years and older in Thailand.

## MATERIALS AND METHODS

### Study design.

**Figure 1.**
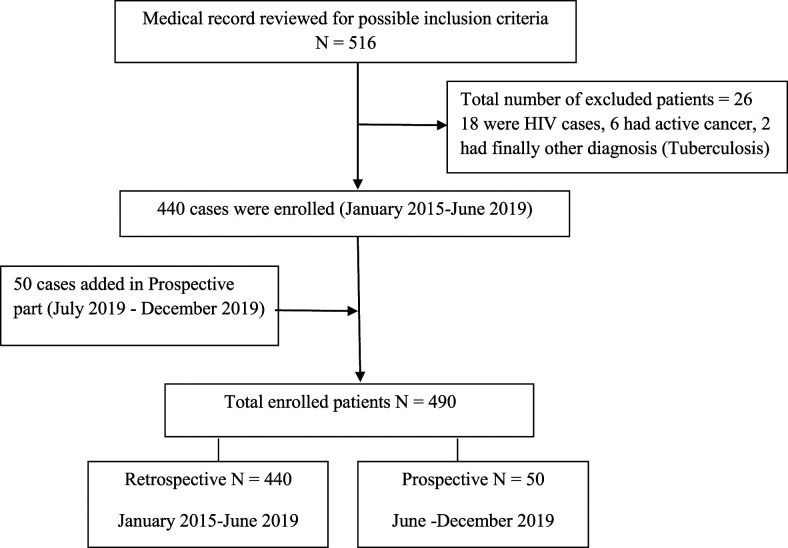
Flowchart of the study.

We performed a retrospective and prospective observational study involving 490 older adult patients (age, 60–107 years) hospitalized with pneumonia from January 2015 to December 2019. The study was conducted at Bamrasnaradura Infectious Diseases Institute in central Thailand (Nonthaburi province), which is a 650-bed general hospital that was originally opened as an infectious diseases institute by the Ministry of Public Health. The majority of patients (440 patients) were included in the retrospective study; however, 50 patients participated in the prospective study (Figure 1). The study protocol was approved by ethics committees of the Faculty of Tropical Medicine, Mahidol University, and Bamrasnaradura Infectious Diseases Institute. All patients participating in the prospective study provided written informed consent.

### Patient eligibility and data collection.

Inclusion criteria were age 60 years or older, no hospitalizations during the previous 14 days, new infiltration observed on chest radiographs, and one or more respiratory symptoms or signs. Because atypical pneumonia presentations are common among this age group, elderly patients with radiological evidence of pneumonia without symptoms were included. Patients were excluded if they were HIV-positive or receiving chemotherapy, or if pneumonia was unconfirmed.

Information regarding the demographic characteristics, signs, symptoms, clinical findings on presentation, comorbidities, laboratory parameters, microbiological/radiological findings, complications during hospitalization, the need for ICU admission, and outcomes after discharge were obtained from medical records. For the prospective study, the same data were collected on admission and patients were observed during their hospital stay. All patients were followed-up for 30 days after discharge from the hospital. A microbiological diagnosis was determined based on the results of culture and polymerase chain reaction (if available) testing of respiratory samples (sputum, pleural fluid, or tracheal aspirates) and blood culture test results.

We used two scoring systems (the CURB-56 score and modified SMART-COP score) to assess disease severity and analyzed the relationships among these scores and ICU admission, the need for intensive respiratory support, and 30-day mortality. The CURB-65 score was calculated based on five variables (confusion, blood urea nitrogen > 7 mmol/L, respiratory rate ≥ 30 beats/min, systolic blood pressure ≥ 90 mmHg, diastolic blood pressure ≤ 60 mmHg, and age older than 65 years; 1 point each). Based on the CURB-65 scores, patients were classified into three risk groups: low (0–1 point), moderate (2 points), or high (3–5 points). Because of the limited laboratory investigation results available in primary care settings in developing countries (e.g., blood gas analysis), we modified the SMART-COP score (systolic blood pressure < 90 mmHg, multilobar infiltration, albumin < 3.5 g/dL, respiratory rate ≥ 30 breath/min, tachycardia > 125 beats/min, new-onset confusion, oxygen saturation ≤ 90%, and blood pH < 7.352) by excluding blood pH as a criterion and named this modified score the SMART-CO score (systolic blood pressure, multilobar infiltration, albumin, respiratory rate, tachycardia, confusion, oxygen saturation). This score assigns 2 points for oxygen saturation ≤ 90% and systolic blood pressure < 90 mmHg, and 1 point for other variables. Patients are accordingly classified into four risk groups: low (0–2 points), moderate (3–4 points), high (5–6 points), and very high (≥ 7 points).

### Statistical analysis.

All data were analyzed using SPSS software version 23 (IBM Corp., Armonk, NY). Categorical variables were summarized and expressed as frequencies and percentages. Quantitative variables were presented as medians and interquartile ranges (IQRs). The χ^2^ test or Fisher’s exact test was used to assess differences between groups as appropriate. The Mann-Whitney *U* test was used to assess differences between non-normally distributed continuous variables. The Kaplan-Meier survival analysis was used to compare survival times between different risk groups. For all analyses, *P* < 0.05 was considered statistically significant.

## RESULTS

### Demographic, clinical, and microbiological characteristics of elderly patients with pneumonia.

Among the 490 patients with pneumonia included in the study, 51.2% were men; the median age was 80 years (IQR, 70–87 years). Most patients (96.9%) were admitted from home or the community; only 15 patients were admitted from nursing homes. Most patients (90.4%) had comorbidities. The most prevalent underlying conditions were hypertension (62.4%), diabetes (35%), neuropsychiatric diseases (17%), and chronic lung diseases (16%). The majority of patients had poor functional status. Most (80.3%) required support from other family members to perform their daily basic activities, and 16.4% were completely bed ridden. Cough, fever, and dyspnea were the most common symptoms of pneumonia. A significant proportion of elderly patients (14.3%) presented with confusion, with a median Glasgow coma scale score of 10 (IQR, 9–12).

Sputum specimens from 92% of patients underwent culture testing. The majority (68.2%) of those sputum samples yielded positive results. Among the positive culture test results, a single bacterial species was detected in 69.5% and multiple bacterial species were detected in 30.5%. *Mycobacterium tuberculosis* was detected in two patients; therefore, those patients were excluded from the study. Blood culture tests were performed for 88% of patients, and only 10.2% had positive results. *Escherichia coli* (8 patients), *Streptococcus pneumoniae* (4 patients), and *Klebsiella pneumoniae* (4 patients) were the most common organisms identified by blood culture tests. Throat swabs were performed for nearly half of the patients (49.3%), and 21.5% had positive results for influenza A virus (34 patients), influenza B virus (13 patients), and respiratory syncytial virus (5 patients). Pleural fluid was obtained from nine patients. A tracheal aspirate was obtained from one patient. Culture test results were positive for *Pseudomonas aeruginosa* for two patients. Most elderly patients with pneumonia (59.3%) had multilobar opacities on chest radiographs.

### Pneumonia etiology, clinical course, and outcomes.

The etiology of pneumonia was known for 72.4% of elderly patients. Bacterial organisms were the most common causes of pneumonia (61.8% of total patients). Other etiologies are shown in Table 1. Gram-negative bacteria were predominant among elderly adults with bacterial pneumonia. *K. pneumoniae* (20.4%) was the most common causative organism, followed by *P. aeruginosa* (15.5%), *Acinetobacter baumannii* (7.3%), *Haemophilus parainfluenzae* (6.7%), and *Haemophilus influenzae* (5.9%). Among Gram-positive bacteria, *Staphylococcus aureus* (8.9%) and *S. pneumoniae* (5.9%) were the most common causative agents of pneumonia. A significant minority (21.9%) of elderly patients with bacterial pneumonia were infected by multidrug-resistant (MDR) or extended-spectrum β-lactamase (ESBL)-producing organisms. ESBL-producing *K. pneumonia*e was the most common etiology, followed by MDR *P. aeruginosa,* ESBL-producing *E. coli*, and MDR *A. baumannii*. Among the 86 patients who died of pneumonia, *K. pneumoniae* was the predominant causative agent (24 patients), followed by *P. aeruginosa* (13 patients) and *A. baumannii* (11 patients). MDR- and ESBL-producing organisms were isolated from 26 elderly patients (30.2%) who died of bacterial pneumonia. ESBL-producing *K. pneumoniae*, MDR *P. aeruginosa*, and MDR *A. baumannii* were the predominant causes of fatal bacterial pneumonia. A total of 113 of 490 patients (23%) were admitted to the ICU for a median duration of 7 days (IQR, 3–14 days), and 80 of 113 patients (70.8%) required mechanical ventilators for a median duration of 7 days (IQR, 4–14 days). Among patients admitted to the ICU, *K. pneumonia*, *P. aeruginosa*, *H. parainfluenzae*, and *S. pneumoniae* were the most common etiological agents. Outcomes were known for 111 of 113 patients admitted to the ICU: almost half (45%) died in the ICU and 55% experienced improvement after ICU admission. The majority (80.8%) of elderly patients with pneumonia survived and were discharged from the hospital after a median duration of hospitalization of 8 days (IQR, 4–16 days), whereas 17.4% of patients died while undergoing care. An analysis of short-term outcomes within 30 days after discharge revealed that mortality rates increased to 19% and 8.7% of discharged patients were readmitted (Table 1).

**Table 1:** Etiology, clinical course, and outcomes of pneumonia among elderly patients (*N* = 490)

	N (%)
Etiology	
Bacterial	303 (61.8)
MDR/ESBL organisms	66/303 (21.8)
Viral	37 (7.5)
Coinfection	15 (3.1)
Unknown	135 (27.5)
Common isolated bacterial organisms	62 (20.4)
*Klebsiella pneumoniae*	47 (15.5)
*Pseudomonas aeruginosa*	22 (7.3)
*Acinetobacter baumannaii*	26 (8.9)
*Staphylococcus aureus*	20 (6.7)
*Haemophilus parainfluenzae*	17 (5.9)
*Haemophilus influenzae*	17 (5.9)
*Streptococcus pneumoniae*	15 (4.9)
*Escherichia coli*	77 (25)
Other GNB	
Complications	153 (31.2)
RDS	66 (13.5)
Plural effusion	92 (18.7)
Septicemia	26 (5.3)
Lung abscess	3 (0.6)
Others	3 (0.6)
Outcome at discharge	
Survived	396 (80.8)
Died	86 (17.9)
Discharged against medical advice	8 (1.6)
Outcome 30 days after discharge	
Cured	203 (41.4)
Improved	32 (6.5)
Readmitted	43 (8.7)
Died	93 (19)
Lost to follow-up	119 (24.2)

MDR/ESBL = multidrug-resistant/extended-spectrum β-lactamase; RDS = respiratory distress syndrome; others = pleurisy, emphysema, and lung collapse; other GNB = *Moraxella catarrhalis*, *Stenotrophomons maltophilia*, *Proteus mirabilis*, *Enterobacter cloacae*, and *Providencia* spp.

### CURB-65 score and SMART-CO score.

**Figure 2.**
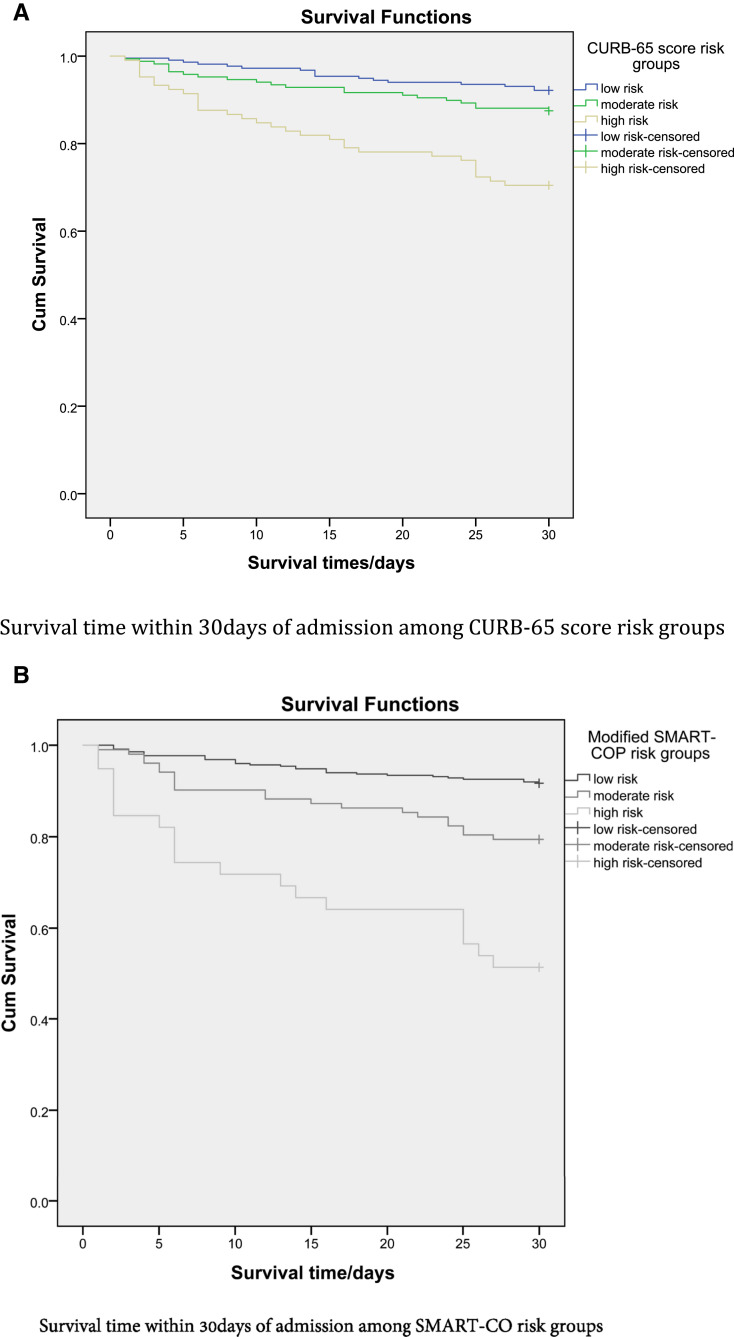
(**A**) Survival time within 30 days of admission among risk groups based on the CURB-65 score. (**B**) Survival time within 30 days of admission among risk groups based on the SMART-CO score. This figure appears in color at www.ajtmh.org.

We retrospectively analyzed the CURB-65 and SMART-CO scoring systems. According to the CURB-65 scores, nearly half of the patients (44.3%) were classified as low risk. Unsurprisingly, 30-day mortality was significantly increased in the high-risk group (*P* < 0.001). When patients were reclassified into two risk groups (low or moderate/high), in-hospital mortality was significantly higher for the moderate/high-risk group (*P* < 0.001; relative risk [RR], 1.7; 95% confidence interval [CI], 1.2–2.4) (Table 4). Using the modified SMART-CO score, more patients (71.2%) were classified as low risk. Patients in higher risk classes had higher rates of ICU admission and required mechanical ventilation more often (*P* < 0.001). All patients in the very high-risk group (score ≥ 7 points) died within 30 days of admission. Patients with SMART-CO scores ≥ 3 (moderate to very high-risk group) had significantly higher in-hospital mortality rates (*P* < 0.001; RR, 1.6; 95% CI, 1.3–2.0) (Table 3). A survival analysis revealed shorter survival times for higher risk groups (*P* < 0.001) when using both scoring systems (Figure 2A and B).

## DISCUSSION AND CONCLUSION

Pneumonia is common for all age groups, but older adults are at increased risk for pneumonia. Pneumonia can cause significant morbidity and mortality, especially in developing countries. Therefore, it is important to understand the etiology and outcomes of pneumonia for individuals at the highest risk.

More than half (51.8%) of the patients with pneumonia in our study were men, which was in agreement with previous studies conducted in Europe^6^ and in Asian countries.^4,23^ Male sex was previously reported as a risk factor for increased pneumonia mortality.^24^ We found that male sex was significantly associated with mortality (*P* = 0.005) (Table 2). Recently, similar trends were observed for coronavirus disease 2019; men were more often affected and had higher mortality rates than women.^25^ Most patients in our study were admitted from the community and only 15 patients were admitted from nursing home, which may indicate that the majority of pneumonia cases among elderly residents in nursing homes in Thailand can be managed without hospital admission. Most patients were partially dependent, and a significant proportion were bedridden, indicating that the majority of elderly patients with pneumonia had poor functional status. Previous studies reported significant associations among poor functional status and short-term and long-term mortality of hospitalized patients with CAP.^26^ This was consistent with our finding that dependence was significantly associated with in-hospital mortality (*P* = 0.012).

**Table 2:** Demographic features and discharge outcomes among elderly patients with pneumonia (*N* = 482/490)

	Recorded (*N* = 482)	Survived (*N* = 396)	Died (*N* = 86)	*P* value
Demographic features				
Male	249 (51.6)	193 (48.7)	56 (65.1)	0.005
Age ≥ 80 years	235 (48.7)	181 (45.7)	54 (62.7)	0.004
Median age	80 (69–87)	79 (68–68)	84 (77–89)	< 0.001
Underlying conditions				
Hypertension	301 (62.4)	248 (62.6)	53 (61.6)	0.765
Diabetes	169 (35)	141 (35.6)	28 (32.5)	0.732
Neuropsychiatric disease	82 (17)	58 (14.6)	21 (24.4)	0.025
Chronic renal diseases	78 (16.2)	60 (15.2)	18 (20.9)	0.402
Chronic lung diseases	65 (13.5)	57 (14.4)	8 (9.3)	0.296
Heart diseases	63 (13)	54 (13.6)	9 (10.5)	0.519
Asthma	18 (3.7)	15 (3.8)	3 (3.5)	0.849
				
				
Physical status				
Bed ridden	79 (16.4)	60 (15.1)	19 (22)	0.115
Partially dependent	357 (74)	284 (71.7)	73 (84.8)	0.012

Outcomes were known for 482 of 490 patients. Eight patients were discharged against medical advice. All values are shown as number (%) of patients except for median age, which is presented as the median (interquartile range).

During this study, pneumonia etiology was known for 72.4% of patients. This rate was higher than that reported previously in some Asian countries, such as Japan, where the etiology was established for 48% of patients,^4^ and in some Western countries.^27,28^ A similar pathogen detection rate (71.4%) was reported among hospitalized patients with CAP in Thailand.^5^ Bacterial infections were the main causes of pneumonia among older adult patients, causing 61.8% of cases. This finding is in general agreement with the findings of previous reports of Asian countries, such as China,^29^ Japan,^4^ and India,^30^ where bacterial infections were implicated in 49%, 59%, and 52% of pneumonia cases, respectively. The rate of older adult patients infected by more than one bacterial pathogen was 29.3%, which was significantly higher than the rate among older adult patients with CAP in Japan (19%).^4^ Multiple bacterial infections were significantly associated with mortality (*P* = 0.001; RR, 1.2; 95% CI, 1.0–1.3), potentially indicating that multiple infections may result in treatment failure unless all organisms are targeted with effective antibiotics. MDR- and ESBL-producing organisms had an important role in the etiology of pneumonia in older adult patients and were significantly associated with in-hospital mortality (*P* < 0.001; RR, 1.3; 95% CI, 1.1–1.6) (Table 3).

**Table 3:** Clinical characteristics and discharge outcomes among elderly patients with pneumonia

	Total (*N* = 482)	Survived (*N* = 396)	Died (*N* = 86)	*P* value	RR (95% CI)
Radiological characteristics					
Multilobar infiltration	286 (59.3)	219 (55.3)	67 (77.9)	< 0.001	2 (1.3–3.0)
Pleural effusion	91 (18.9)	61 (15.4)	30 (34.9)	< 0.001	1.2 (1.1–1.5)
RDS	62 (12.9)	28 (7.1)	34 (39.5)	< 0.001	1.5 (1.2–1.8)
					
Clinical course					
ICU admission	111 (23.0	61 (15.4)	50 (58.0)	< 0.001	1.8 (1.4–2.3)
Ventilator use	78 (16.2)	36 (9.1)	42 (48.8)	< 0.001	1.7 (1.4–2.2)
Pneumonia complication	143 (29.7)	84 (21.2)	59 (68.6)	< 0.001	2.5 (1.8–3.4)
Etiology					
MDR/ESBL organisms	66 (13.7)	42 (10.6)	24 (27.9)	< 0.001	1.3 (1.1–1.6)
Multiple bacterial infections	92 (19.1)	65 (16.4)	27 (31.4)	< 0.001	1.2 (1.0–1.3)
SMART-CO score					
Moderate to very high-risk group (≥ 3 points)	136 (28.2)	90 (22.7)	46 (53.5)	< 0.001	1.6 (1.3–2.0)
CURB-65 score					
Moderate to high-risk group (≥ 2 points)	268 (55.6)	206 (52)	62 (72)	< 0.001	1.7 (1.2–2.4)
					

RR = risk ratio; 95% CI = 95% confidence interval; RDS = respiratory distress syndrome; MDR/ESBL = multidrug-resistant/extended-spectrum β-lactamase; ICU = intensive care unit. All values are shown as the number (%) of patients.

**Table 4:** CURB-65 and SMART-CO scores on admission, clinical course, and 30-day mortality among elderly patients with pneumonia

Risk group (*N* = 490)	ICU admission	Ventilator use	30-day mortality
CURB-65 score			
Low risk (0–1 points) 217 (44.3)	26 (11.9)	16 (7.4)	17 (7.8)
Moderate risk (2 points) 168 (34.3)	37 (22.0)	26 (15.5)	21 (12.5)
High risk (3–5 points) 105 (21.4)	50 (47.6)	38 (36.2)	31 (29.5)
*P* value	< 0.001	< 0.001	< 0.001
SMART-CO score			
Low risk (0–2 points) 349 (71.2)	56 (16.0)	36 (10.3)	29 (8.3)
Moderate risk (3–4 points) 102 (20.9)	37 (36.3)	26 (25.5)	21 (20.6)
High/very high risk (≥ 5 points) 39 (7.9)	20 (51.2)	18 (46.2)	19 (48.7)
*P* value	< 0.001	< 0.001	< 0.001

ICU = intensive care unit. All values are shown as the number (%) of patients.

Several studies have demonstrated that Gram-negative bacteria are uncommon causes of CAP,^24^ and that *S. pneumoniae* remains the most frequently identified causative agent of CAP in Western European countries^31^ as well as in older adult patients hospitalized with CAP in some Asian countries, including Thailand.^4^ However, our study emphasizes the importance of Gram-negative bacteria as a common cause of CAP in the elderly. We found that *K. pneumoniae* (20.4%), *P. aeruginosa* (15.5%), and *A. baumannii* (7.3%) were the most common causative organisms. These organisms were identified at lower frequencies among hospitalized adults with CAP in general hospitals in Thailand; however, previous studies included all adults. We focused on older adults, suggesting that infection by these organisms was more frequent among the older adults. This is in agreement with the findings of another study conducted in Thailand that showed that Gram-negative infection was more likely to occur in elderly patients with comorbidities.^5^ Similar rates of these organisms (*K. pneumoniae*, 21.3%; *P. aeruginosa*, 17.3%; *A. baumannii*, 10.7%) were observed in elderly patients with severe CAP in Taiwan,^9^ potentially indicating that Gram-negative bacteria are a common cause of pneumonia among the elderly in this region. Among Gram-positive bacteria, the prevalence of *S. pneumoniae* infection was lower than that in Thailand and overseas.^32^ Conversely, the *S. aureus* prevalence was higher than that previously reported for hospitalized adults with CAP in Khon Kean in north Thailand,^33^ but it was similar to that observed among elderly patients with severe pneumonia in Taiwan. Among elderly patients admitted to the ICU, *K. pneumoniae* was the most common causative agent of pneumonia. This finding is compatible with previous data of patients with severe pneumonia admitted to ICUs in Singapore.^34^ In the ICU, mortality rates were significantly higher for patients with *S. aureus*, *E. coli*, *A. baumannii*, and *K. pneumoniae* infections. A previous study of ICUs showed similar results.^9^

Viral pneumonia was less frequent and appeared to be less severe, resulting in no ICU admissions or deaths. We found that viruses were responsible for 7.5% of pneumonia cases among the elderly; this rate was less than those reported for China,^29^ Japan,^4^ and the Philippines^8^ (26.9%, 13%, and 13% of pneumonia cases, respectively). This finding may be related to the limited use of molecular techniques for the identification of respiratory viruses in our study. However, it is in line with the finding of a previous study performed in Thailand that showed that viral pneumonia was less common in elderly patients and occurred more frequently in younger age groups (19–40 years).^2^ Our study was completed 1 month before the rapid spread of the novel severe acute respiratory syndrome coronavirus-2.

In-hospital mortality during our study was 17.6%, and it was as high as 45% for patients admitted to the ICU. Mortality rates for elderly patients hospitalized with CAP reported by previous studies have ranged from 6% to 40%.^1,24,35,36^ Our findings are similar to those of previous studies conducted overseas^37,38^ that reported that in-hospital mortality for elderly patients with pneumonia ranged from 15.0% to 15.3%. They are also similar to previous data from Thailand indicating a mortality rate of 15.5%.^2^ Within 30 days after discharge, the rate of readmission was 8.7%, which was compatible with the results of previous studies.^14,15^

Assessing the severity of pneumonia is an important step in the clinical management of elderly patients. Two scoring systems were used to assess severity among elderly patients with pneumonia (CURB-65 and SMART-CO). Compared with the CURB-65 score, a higher proportion of elderly patients were classified as the low-risk group by the SMART-CO score. This is because the original SMART-COP score was based on disease severity and did not consider underlying risk factors such as age. During our study, patients with higher risk classes had higher rates of ICU admission, were more likely to require intensive respiratory support, and had significantly increased 30-day mortality (Figure 2). Identification of high-risk patients using these scores in resource-limited settings with limited ICU beds can help inform appropriate management strategies and potentially improve patient outcomes.

In conclusion, our study highlighted that Gram-negative bacteria are a common cause of pneumonia in older adult patients. Infection by Gram-negative bacteria should be suspected in elderly patients with coexisting illnesses and poor functional status. Elderly individuals with pneumonia accompanied by multiple comorbidities likely require ICU admission. Gram-negative bacterial infections should also be considered in ICU settings and in intubated patients. Pneumonia mortality remains high, especially in elderly patients admitted to ICUs. Both the CURB-65 and SMART-CO scores showed similar trends of higher rates of ICU admissions, ventilator use, and 30-day mortality for high-risk groups (*P* < 0.001). Further studies should examine associations between Gram-negative bacterial infections and severe pneumonia among the elderly. Larger prospective studies could be better able to assess the sensitivity and specificity of the SMART-CO score for predicting the need for ICU admission and intensive respiratory support among elderly patients with pneumonia.
